# Navigation-Assisted Bilateral Troughing Technique for Lumbar Laminectomy: A Stepwise Approach to Safe, Teachable, and Efficient Decompression

**DOI:** 10.7759/cureus.109651

**Published:** 2026-05-25

**Authors:** Imran Siddiqi, Allison Shay, Arisa Ueno, Stanley Dennison, Christopher Wong, Sanjay C Rao, Raed Sweiss, Dan E Miulli

**Affiliations:** 1 Neurosurgery, Riverside University Health System Medical Center, Moreno Valley, USA; 2 Anatomical Sciences, St. George's University School of Medicine, True Blue, GRD; 3 Neurological Surgery, Kaiser Permanente Fontana Medical Center, Fontana, USA

**Keywords:** diamond burr, en bloc, facet preservation, intraoperative navigation, lumbar laminectomy, lumbar stenosis, matchstick burr, spinal decompression, troughing technique

## Abstract

Lumbar laminectomy is one of the most common spinal procedures, yet the extent of bone removal remains highly operator-dependent, resulting in significant variability in decompression outcomes. Insufficient decompression risks symptom persistence, while over-resection of the facet joints causes iatrogenic instability, and uncontrolled instrument passes near the inner lamina cortex carry well-documented risk of dural injury and cerebrospinal fluid (CSF) leak. A standardized, stepwise technique that reduces these risks, promotes operative efficiency, and provides a teachable framework for neurosurgical residents has not been widely described. We present a reproducible six-step lumbar laminectomy technique centered on bilateral troughing with a 3-mm matchstick burr, progressive eggshell thinning with a 5-mm diamond burr, and en bloc lamina removal, with intraoperative navigation used at defined checkpoints to confirm the medial facet border, pedicle proximity, trough depth, and final decompression extent. The technique is illustrated with a case of a 70-year-old man with multilevel lumbar stenosis (Schizas grade C-D at L3-5) and a prior L4-5 discectomy who underwent L3-L5 laminectomy using this approach; the estimated blood loss was 30 mL, the troughing and en bloc removal were completed in approximately 15 minutes, and the patient was discharged on postoperative day three without complication. The staged matchstick-to-diamond burr sequence reduces the risk of inadvertent dural injury while maintaining operative efficiency, and navigation serves as a confirmatory adjunct rather than a primary guide. The technique is designed to be learned by neurosurgical residents, with each step building anatomical understanding and safeguarding against dural injury and facet violation, and merits prospective evaluation in larger cohorts.

## Introduction

Lumbar laminectomy is one of the most frequently performed procedures in spinal surgery, serving as the primary surgical treatment for symptomatic degenerative lumbar canal stenosis. The goals of the operation are well established: adequate decompression of the neural elements through removal of the posterior arch, ligamentum flavum, and any contributing hypertrophied facet tissue, while preserving the stability conferred by the facet joints and paraspinal musculature. In practice, achieving adequate decompression frequently requires partial medial facetectomy to address lateral recess stenosis and foraminotomy to relieve foraminal compression, in addition to the central laminectomy. Despite the frequency with which the procedure is performed, significant variability persists in how these goals are achieved, particularly in the extent of bony removal, the definition of decompression endpoints, and the degree of facet preservation [1].

This variability is not without clinical consequence. Inadequate decompression, whether from underthinning of the lamina, incomplete flavectomy, or failure to adequately address the lateral recess, may leave the patient with persistent neurogenic claudication or radiculopathy and predispose to early reoperation. Conversely, excessive resection of the facet joints, particularly bilateral partial or complete facetectomy, has been demonstrated in cadaveric and finite-element studies to significantly increase segmental motion and predispose to mechanical instability [1,2]. Additionally, uncontrolled or misdirected instrument passes near the inner cortex carry a well-documented risk of iatrogenic dural injury and cerebrospinal fluid (CSF) leak. Reported incidences range from 2.3% in primary cases to over 16% in revision surgery [3,4]. These outcomes impose a substantial burden on patients and the healthcare system, underscoring the importance of a technique that achieves reliable, reproducible decompression within clearly defined anatomical boundaries. Incomplete lateral recess decompression and inadequate undercutting of the medial facets represent the most commonly cited causes of persistent or recurrent radiculopathy following lumbar decompression, frequently necessitating revision surgery. This is a burden that carries substantial operative risk, prolonged disability, and added healthcare cost. Navigation-assisted techniques that provide objective, real-time confirmation of decompression extent are therefore not merely a technical refinement but a direct response to a well-documented source of surgical failure.

Intraoperative navigation has transformed the accuracy and reproducibility of pedicle screw placement across cervical, thoracic, and lumbar spine surgery [5]. Despite this, its application to decompressive procedures has been comparatively underexplored. Navigation offers real-time, three-dimensional anatomical localization that can define the critical direction and boundaries of decompression. Most importantly, the medial edge of the facet complex is well defined and confirmed, particularly in cases where there are significant degenerative changes or facet overgrowth. Population-level data demonstrate that reoperation following lumbar decompression occurs in approximately 11% of patients at 10-year follow-up, driven in substantial part by inadequate initial decompression, which underscores the importance of a technique that provides objective, real-time verification of decompression boundaries at the time of the index operation [6]. These data suggest that current standard techniques leave meaningful room for objective improvement in decompression verification.

The removal of the lamina as a single structural unit following bilateral trough creation has precedent in oncologic spinal surgery and laminoplasty, and has been applied to degenerative stenosis in limited series with favorable results [7]. Our group previously described a handheld osteotome approach to single-unit laminectomy, validating the technique in a cadaveric model and demonstrating its clinical application in an illustrative case, establishing the feasibility and safety of non-piecemeal posterior arch removal [8]. By removing the posterior arch as one piece rather than through repeated piecemeal passes with Kerrison rongeurs, en bloc techniques offer improved structural control, consistent thecal sac visualization, and potentially reduced operative time. The adoption of a staged burr strategy beginning with an efficient matchstick burr for trough creation and transitioning to a diamond burr for delicate inner cortical thinning provides a safety framework analogous to that used in skull base neurosurgery, tailored here for the lumbar spine.

We describe a stepwise technique integrating these principles: bilateral longitudinal troughing with a matchstick burr, progressive eggshell thinning and completion with a diamond burr, en bloc lamina removal, and intermittent intraoperative navigation to confirm the surgical direction and anatomical boundaries at defined checkpoints. The technique is described in full in the case presentation below, as applied in a single illustrative case. We present the technique and case as a structured operative description rather than a comparative outcome study, with the explicit recognition that prospective evaluation in larger cohorts is required to establish its decompression adequacy, efficiency, and complication profile relative to conventional techniques.

## Case presentation

A 70-year-old man was referred for the surgical evaluation of progressive lumbar spinal stenosis. The patient reported a 15-year history of low back pain with bilateral lower extremity radiculopathy, progressive in severity over recent years. Symptoms included a burning dysesthesia in an L4-L5 distribution bilaterally, radiating from the lateral thighs to the posterior calves, worse on the right than the left. He described neurogenic claudication limiting ambulation to less than one block before symptom onset, consistently aggravated by prolonged standing or walking and relieved by sitting or lumbar flexion. Pain intensity was reported at 7/10 and had severe functional impairment. He denied saddle anesthesia, bowel or bladder dysfunction, or significant lower extremity weakness.

The patient had an extensive non-surgical treatment history spanning multiple years, including physical therapy, epidural steroid injections, oral non-steroidal anti-inflammatory drugs (NSAIDs), muscle relaxants, and gabapentin. Injections had previously provided partial and temporary relief but were no longer effective. He reported a prior partial L4-5 discectomy performed at an outside institution several years earlier. This prior surgery was a key determinant in the preoperative navigation plan: the expected scar tissue and altered tissue planes at L4-5 made navigation-assisted trough confirmation particularly valuable, as direct tactile and visual assessment of the medial facet border can be unreliable in revision anatomy. Navigation allowed the neurosurgical trainee to have objective identification of the trough lines despite the distorted posterior element anatomy.

Neurological examination demonstrated diminished sensation in the L4 and L5 dermatomes bilaterally. Motor strength was preserved in all myotomes of the bilateral lower extremities. Straight leg raise testing was positive bilaterally, reproducing the patient's characteristic radicular symptoms. Reflexes were symmetric. Gait assessment revealed a cautious, slow-paced pattern consistent with neurogenic claudication, without frank antalgic posturing.

Preoperative magnetic resonance imaging of the lumbar spine without contrast demonstrated multilevel degenerative disease with 3-mm anterolisthesis at L4-5 (Figure 1A, B). Disc space narrowing with osteophyte formation and Modic type II fatty degenerative endplate changes were identified at L4-5 and L5-S1, with additional Modic type I edema at L4-5. At L3-4, severe spinal canal stenosis was present with ligamentum flavum hypertrophy, diffuse disc bulge, and facet arthropathy, resulting in cauda equina bundling; the dural sac cross-sectional area (DSCA) measured 67 mm² (Schizas grade D, the most severe category of the Schizas morphological grading scale, denoting no recognizable rootlets within the dural sac on axial T2-weighted imaging). At L4-5, moderate-to-severe stenosis was identified with an uncovered disc, diffuse disc bulge, facet arthropathy, and moderate bilateral foraminal compromise; DSCA measured 88.2 mm² (Schizas grade C). Flexion-extension radiographs demonstrated no significant dynamic change in the 3-mm anterolisthesis at L4-5, confirming the absence of dynamic instability at that level.

**Figure 1:**
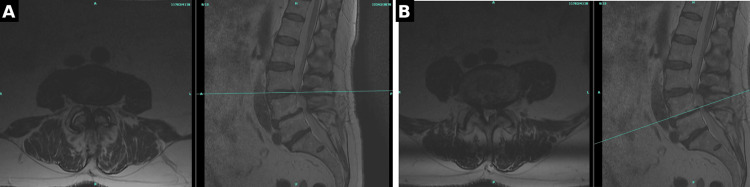
Preoperative MRI demonstrating multilevel lumbar stenosis (A) Side-by-side axial and sagittal reformats at L3-4 confirming the level and extent of maximal stenosis for operative planning; severe spinal canal stenosis (Schizas grade D) with ligamentum flavum hypertrophy, facet arthropathy, diffuse disc bulge, and cauda equina bundling, with dural sac cross-sectional area (DSCA) of 67 mm². (B) Side-by-side axial and sagittal reformats at L4-5 demonstrating moderate-to-severe stenosis (Schizas grade C) with uncovered disc, facet arthropathy, and bilateral foraminal compromise; DSCA 88.2 mm². DSCA, dural sac cross-sectional area.

Given the severity of multilevel stenosis, failure of extensive nonsurgical management, and progressive functional decline, the patient was counseled on and consented to L3-L5 laminectomies using the navigation-assisted troughing technique described below. Fusion was not performed as the flexion-extension radiographs confirmed the absence of dynamic instability, and this technique’s facet-preserving approach maintains posterior column stability without instrumentation.

Operative technique

Patient Positioning and Exposure

The patient is positioned prone on a radiolucent Jackson table or equivalent frame. All pressure points are carefully padded, and the abdomen is allowed to hang freely to minimize epidural venous engorgement and reduce intraoperative blood loss. A standard midline skin incision is made over the operative level(s), and subperiosteal dissection is performed bilaterally to expose the spinous processes, laminae, and the medial aspects of the facet joints. Exposure is extended sufficiently lateral to clearly visualize the facet capsule without violating it. The operative level is confirmed with intraoperative fluoroscopy or navigation prior to beginning any bony work.

Navigation Setup and Registration

Following exposure, a reference array is secured to a stable bony structure at a non-operative level, typically the spinous process one level above the planned decompression. Three-dimensional intraoperative imaging is then acquired using the available platform (isocentric C-arm, O-arm intraoperative CT, or visible-light surface navigation system). Registration accuracy is verified against palpable anatomical landmarks, including the spinous process tips and medial facet borders, before any navigated instruments are used.

At this baseline stage, axial and sagittal navigation views are reviewed to identify: (1) the medial border of facet complex process bilaterally at each operative level, which defines the lateral limit of safe decompression; (2) the location of the pars interarticularis; and (3) the cranio-caudal extent of the stenotic segment requiring decompression. This planning step takes approximately two to three minutes and establishes the anatomical roadmap for the remainder of the procedure.

In the illustrative case, an optical surface-capture navigation system was selected as the intraoperative navigation platform. The operative field was prepared and draped in a sterile fashion, and a midline incision was made with subperiosteal exposure of the posterior elements from L3 to L5. A dynamic reference array was anchored to the spinous process of L2, one level cranial to the planned decompression, using a bone-anchored clamp, and confirmed to be rigidly fixed prior to registration. Following exposure, the optical surface-capture navigation system was registered using the manufacturer’s surface-capture protocol (Figure 2). Registration accuracy was confirmed against palpable anatomical landmarks, including the spinous process tips and the medial facet borders at L3, L4, and L5.

**Figure 2:**
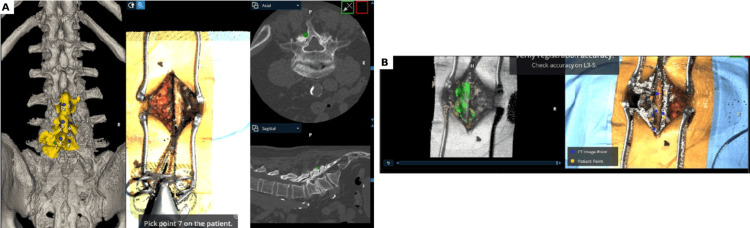
Intraoperative navigation registration using visible-light surface capture (A) Intraoperative screenshot of the navigation interface at the time of system initialization, demonstrating real-time anatomical overlay. (B) Surface registration capture showing the green point cloud generated by the optical registration system, confirming accurate surface matching to the preoperative imaging dataset without ionizing radiation.

Step 1: Lateral Boundary Confirmation

Before any drilling is initiated, a navigated probe is used to confirm the medial edge of the facet complex on each side at the operative level. This landmark defines the lateral boundary of each planned trough. The surgeon confirms that the intended trough lines lying just medial to the facet capsule are correctly positioned relative to this boundary. This is the primary safety checkpoint of the procedure: establishing the lateral limit of decompression prior to any bone removal ensures that subsequent drilling does not encroach on the facet joint.

In straightforward cases with preserved anatomy and normal body habitus, this confirmation typically reinforces what direct visualization already suggests. In more complex cases, multilevel decompression, post-fusion anatomy, high body mass index, or revision surgery with altered tissue planes, navigation provides objective confirmation that cannot be reliably obtained by tactile assessment alone.

In the illustrative case, navigation screenshots were obtained in an axial view to identify and confirm the medial border of the superior articular process at L3, L4, and L5 bilaterally, defining the planned lateral limits of the bilateral troughs (Figure 3). A navigated probe was used to confirm trough positions at each level prior to initiating drilling (Figures 3E,F).

**Figure 3:**
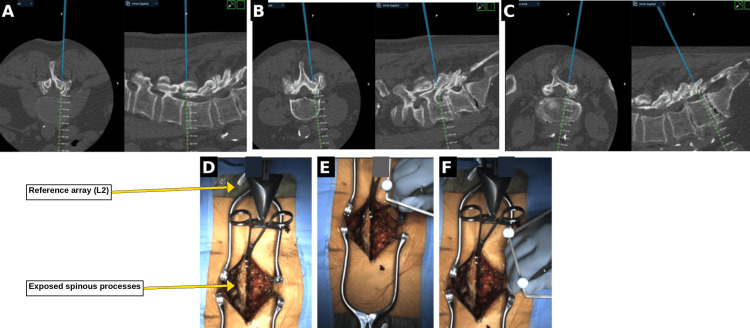
Preoperative trough planning and navigation-confirmed anatomical boundaries (A-C) Navigation screenshots in axial view confirming the planned bilateral trough positions at L3 (A), L4 (B), and L5 (C), with the medial border of the facet complex identified as the lateral limit of safe decompression at each level. (D) Intraoperative photograph of the operative field following registration, demonstrating midline exposure prior to any bony resection. (E) Navigated probe confirming the planned trough position at L3, with the probe tip positioned just medial to the facet capsule. (F) Navigated probe confirming the planned trough position at L5 prior to initiating drilling.

Step 2: Bilateral Trough Creation with Matchstick Burr 

A high-speed matchstick (side-cutting) burr (3 mm) is selected for initial trough creation. The matchstick burr is well-suited to this step due to its efficient lateral cutting profile, which allows a rapid creation of a defined longitudinal channel directed along the planned trough lines without the axial penetration risk associated with end-cutting instruments. Notably, a single-level unilateral trough can typically be created in under two minutes per level with a 3-mm matchstick burr, minimizing cumulative heat exposure to the adjacent neural elements. An alternative to the matchstick burr for trough creation is the bone scalpel (ultrasonic osteotome), which cuts efficiently with lower thermal output. However, the bone scalpel carries its own learning curve and risk of dural injury if advanced too slowly or if the instrument plunges unexpectedly through thinned bone. The matchstick-to-diamond burr sequence described here is a natural neurosurgical instrument set, readily available in any spine operating room, and is specifically chosen for its applicability to resident training: the tactile and visual feedback of progressive bone thinning builds operative anatomy skills that complement formal didactic education. Continuous irrigation with normal saline is maintained throughout all drilling to dissipate frictional heat, as thermal thresholds for bone necrosis and neural injury can be reached with sustained high-speed drilling in the absence of adequate cooling [9].

Beginning at the cranial or caudal end of the operative level on one side, the matchstick burr is used to create a longitudinal trough just medial to the facet joint, advancing in the sagittal plane, proceeding in a caudal-to-cranial direction. This direction of advancement keeps the surgeon working away from the already-exposed dura inferiorly and allows the ligamentum flavum attachment at the caudal laminar edge to serve as an initial protective reference point. The trough is developed to progressively reduce laminar thickness from the outer cortex inward, stopping well short of the inner cortex. The ligamentum flavum is deliberately preserved at this stage, serving as a protective layer between the drilling instrument and the underlying dura and neural elements. The process is then repeated on the contralateral side, creating symmetric bilateral troughs at each operative level (Figure 4). This step is intentionally designed to be teachable, especially for junior neurosurgery residents. The resident neurosurgeon begins by drilling with the 3-mm matchstick burr under supervision, learning the feel of outer cortical bone and the visual landmarks that define the trough line. The senior resident or attending may take over for the final inner cortical thinning with the diamond burr until the junior resident has developed the requisite tactile discrimination and anatomical decision-making.

**Figure 4:**
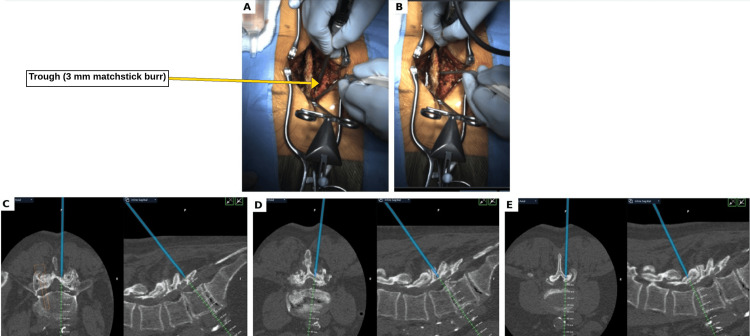
Bilateral longitudinal trough creation with matchstick burr and real-time navigation confirmation. (A) Intraoperative photograph demonstrating matchstick burr trough creation at L5, with continuous saline irrigation to dissipate frictional heat. The burr advances just medial to the facet capsule in a caudal to cranial direction. (B) Intraoperative photograph of matchstick burr trough creation at L3. The ligamentum flavum is preserved intact at this stage, serving as a protective layer between the drilling instrument and the underlying neural elements. (C) Navigation screenshot (axial view) during trough creation at L5, confirming the lateral extent of the burr remains medial to the facet joint. (D) Navigation axial view during trough creation at L4, demonstrating trough trajectory and relationship to the facet joint. (E) Navigation axial view during trough creation at L3, confirming trough positioning relative to the medial facet borders.

Navigation is used intermittently during trough creation to confirm that the lateral extent of the burr does not encroach on the medial facet border or pedicle, is directed away from the dura, and that the depth of the trough does not violate the inner cortical layer. Axial navigation views provide a useful cross-check against direct tactile assessment of remaining laminar thickness. Critically, the lateral placement of the trough line at this step is the primary determinant of facet complex and pars interarticularis preservation for all subsequent steps. The trough must remain medial to the medial facet border; any inadvertent lateral drift at this stage is far more easily recognized and corrected during trough creation than after the lamina has been elevated. Navigation confirmation that the burr has not encroached beyond approximately one-third (30%) of the articular surface should be performed before proceeding, as the pars interarticularis and facet joint capsule cannot be reconstructed once violated [1,10-12].

In the described case, bilateral longitudinal troughs were created at L3, L4, and L5 using a high-speed matchstick burr, advancing from caudal to cranial just medial to the facet capsule at each level under continuous saline irrigation (Figure 4A-E). Navigation confirmation was performed intermittently at each level to verify the trough depth and lateral boundary (Figure 4C-E).

Step 3: Eggshell Thinning With Diamond Burr

Once bilateral troughs have been established and the lamina has been thinned to a cortical shell, the matchstick burr is exchanged for a diamond burr. The diamond burr (5 mm) is selected for this final thinning phase because of its characteristically different cutting behavior: the fine abrasive surface removes bone in a controlled, progressive manner and does not carry the snag-and-plunge risk that can result in inadvertent dural penetration when a cutting burr is advanced in close proximity to the inner cortex of the lamina [13]. This two-burr approach mirrors the strategy routinely used in posterior fossa surgery and skull base decompression, adapted here for the lumbar posterior arch.

The diamond burr is used to thin the remaining cortical shell bilaterally until the lamina achieves an eggshell consistency, which is thin enough to flex under gentle pressure, with the underlying ligamentum flavum visible or palpable through the bone. A seasoned neurosurgeon may choose to complete the trough entirely through the inner cortex with the diamond burr in a single pass, relying on well-developed tactile feedback to avoid dural entry. For surgeons in training or in complex anatomy, the eggshell-first approach described here is preferred: it maintains a thin cortical buffer until controlled blunt elevation is performed, reducing the risk of inadvertent canal penetration. A critical intraoperative landmark during this phase is the visual and tactile transition from cancellous to inner cortical bone in a direction away from the dura. As the diamond burr works cranial-caudal through the cancellous layer, the bone surface appears red and actively bleeding due to the rich vascularity of the trabecular marrow. As the inner cortical layer is reached, this bleeding characteristically diminishes and the bone surface transitions to a denser, paler, avascular appearance. This is a reliable visual cue that the final cortical shell has been encountered and that the dura lies in close proximity. At this point, the surgeon should reduce the drilling pressure, rely on the lightest touch of the diamond burr, and transition to navigation confirmation. Intraoperative navigation in the axial view provides an objective measure of residual bone thickness and canal proximity and direction, serving as a complementary checkpoint to this visual transition. Recognizing this two-stage appearance of vascular cancellous bone followed by the pale, avascular inner cortex, is the key to achieving consistent eggshell thinning without inadvertent canal entry.

A navigation checkpoint is performed at the conclusion of this step to confirm adequate trough depth bilaterally across the full cranio-caudal extent of the planned resection, and to verify that the inner cortex has been sufficiently thinned without canal violation. In the illustrative case, the matchstick burr was exchanged for a diamond burr, which was used to complete inner cortical thinning at each level to an eggshell consistency (Figure 5A-G). Navigation screenshots confirmed canal integrity at each level prior to lamina elevation (Figure 5A,C,E).

**Figure 5:**
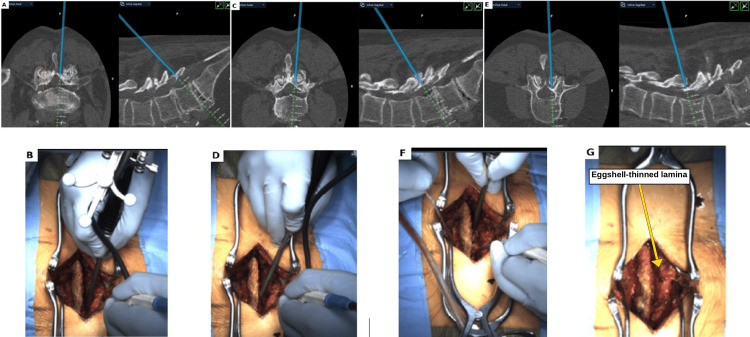
Progressive eggshell thinning of the posterior arch using a diamond burr (A, B) Navigation screenshot and corresponding intraoperative photograph at L5 during diamond burr thinning, with the ligamentum flavum visible through the thinned bone. (C, D) Navigation screenshot and operative photograph at L4 confirming symmetric trough depth and inner cortical thinning. (E, F) Navigation screenshot and operative photograph at L3, completing the eggshell thinning sequence. At each level, the navigation axial view confirms canal integrity prior to en bloc elevation. (G) Intraoperative close-up view of the completed eggshell thinning demonstrating the thin uniform cortical shell remaining prior to en bloc removal.

Step 4: Lamina Removal

With both troughs completed and the lamina reduced to an eggshell construct, en bloc removal is initiated. The interspinous ligament is cut at the cranial and caudal ends of the laminectomy using heavy Mayo scissors, taking care not to inadvertently cut the thecal sac. The spinous process is grasped firmly with a bone-holding clamp such as a Kocher clamp. An upgoing curette or equivalent blunt dissector is introduced between the inner cortical surface and the underlying ligamentum flavum at the midpoint of the operative level, and the plane is developed gently in both cranial and caudal directions. The eggshell lamina separates along the trough lines with controlled upward traction, and the entire posterior arch, including the spinous process and bilateral laminae, is removed as a single construct.

The lamina elevation removal step is made possible by the precision of the bilateral troughs created in Steps 2 and 3. The trough geometry constrains the fracture plane, ensuring that separation occurs along the intended lines rather than propagating unpredictably across the lamina. The primary advantage of this step is that it avoids repeated blind passes of a Kerrison rongeur through a compromised canal; however, the troughing technique itself is the critical technical contribution, as it creates the controlled fracture planes that make safe, single-unit removal possible.

For the patient described, the superior and inferior ligamentous attachments of the posterior arch were released with a rongeur at the L2-3 and L5-S1 junctions, respectively (Figure 6A). A curette was used to develop the plane between the inner cortical surface and the underlying ligamentum flavum (Figure 6B). With bilateral Kocher clamps applied to the superior and inferior extents of the construct, the L3-L5 posterior arch was elevated en bloc along the trough fracture planes and removed as a single unit (Figure 6C-E).

**Figure 6:**
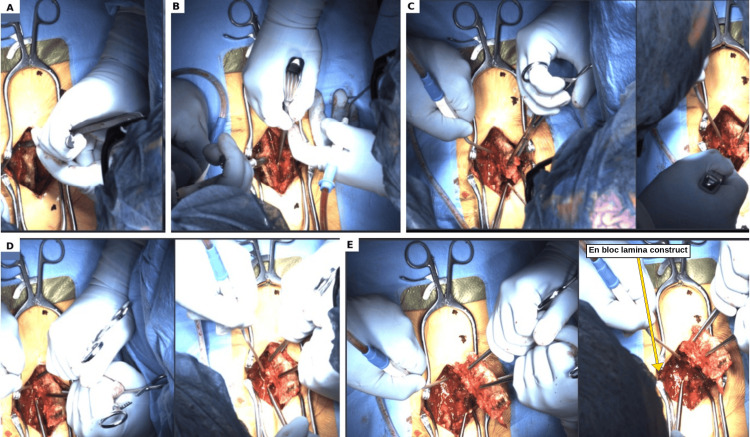
Sequential steps of lamina removal at L3–L5 (A) A rongeur severs the ligamentous and bony attachment superiorly at L2-3 and inferiorly at L5-S1, freeing the cranial and caudal extents of the construct. (B) A curette introduced beneath the lamina with gentle upward pressure detaches the ligamentum flavum from the undersurface of the laminae. (C) The inferior aspect of the L5 lamina is elevated, demonstrating controlled separation along the trough fracture plane. (D) Two Kocher clamps applied to the superior and inferior edges of the laminar construct allow simultaneous controlled elevation. (E) Completed en bloc removal of the L3-L5 posterior arch as a single unit, providing immediate wide exposure of the underlying dura and ligamentum flavum.

Step 5: Ligamentum Flavum Resection and Lateral Recess Decompression

Following lamina removal, the ligamentum flavum is directly visualized and resected using a Woodson elevator or Penfield dissector to protect the underlying dura from the Kerrison rongeurs, working from the midline laterally on each side. Care is taken to identify and preserve the dural sleeve and nerve root as the flavum is removed. Lateral recess decompression is then performed as required, undercutting the superior articular process with a diamond drill or a protected small-bite Kerrison rongeur to relieve foraminal and lateral recess compression. A diamond burr may be used to undercut the medial wall of the pedicle and widen the foramen under direct visualization, followed by sequential Kerrison passes to complete foraminal decompression. Safety is paramount during this step: the diamond burr should be used with light, controlled movements in the cranial caudal direction and constant irrigation; aggressive lateral drilling risks encroachment on the pedicle and exiting nerve root. Working quickly and deliberately with short, intermittent bursts of drilling minimizes thermal exposure. Navigation is used throughout this step to confirm that undercutting of the articular process does not extend beyond approximately one-third (30%) of the articular surface bilaterally. Preservation of the pars interarticularis is equally critical: resection beyond 50% of the pars significantly increases the risk of iatrogenic spondylolisthesis. Notably, the anatomic window available for safe decompression varies by level. The ratio of pars interarticularis distance to spinal canal width is narrower at L1-L3 compared with L4-L5, meaning that the same absolute extent of bony resection carries greater relative risk at upper lumbar levels [14]. Facetectomy should be limited to no more than approximately one-third (30%) of the articular surface; resection beyond this threshold produces progressive increases in segmental motion, intradiscal pressure, and facet loading, creating conditions favorable for instability [1,10-12]. Foraminal adequacy is confirmed by the passage of a nerve hook or Woodson freely around the exiting nerve root without resistance, and by direct visualization of a freely pulsating dural sleeve at the foramen.

In the illustrative case, the ligamentum flavum was subsequently resected, and bilateral foraminotomies and lateral recess decompression were performed using a high-speed diamond burr and Kerrison rongeurs in the foramina (Figure 7A,B). The final decompressed thecal sac was visualized pulsating freely without dural defect (Figure 7C). The en bloc lamina specimen was documented (Figure 7D).

**Figure 7:**
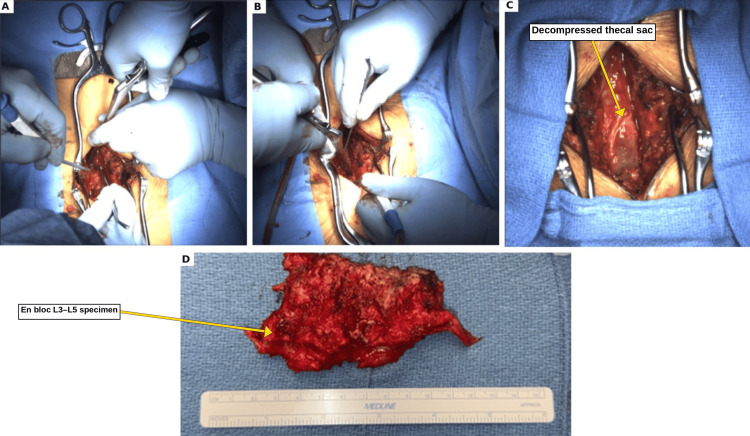
Final decompression, neural element visualization, and en bloc lamina specimen (A) Lateral recess decompression and foraminotomy using a Kerrison rongeur bilaterally, with a Penfield dissector protecting the thecal sac and traversing nerve roots. (B) Ligamentum flavum resection using a Kerrison rongeur with the Penfield dissector maintaining a protective plane between the instrument and the underlying dura. (C) Final intraoperative view of the decompressed thecal sac following laminectomy and flavectomy at L3–L5, demonstrating wide posterior decompression with the dura pulsating freely and no dural defect identified. The bilateral facet joints are preserved at the lateral margins of the decompression field. (D) The en bloc lamina specimen following removal, demonstrating single-unit resection of the L3 L5 posterior arch including the spinous processes and bilateral laminae. The geometry of the specimen reflects the bilateral trough fracture planes and confirms symmetric, controlled resection.

Step 6: Final Navigation Verification and Wound Closure

At the completion of decompression, a final navigation verification is performed. Axial and sagittal reconstructions are reviewed to confirm: (1) adequate lateral decompression to the level of the medial pedicle wall; (2) bilateral facet preservation above the 30% threshold; and (3) cranio-caudal decompression spanning the full extent of the planned resection. Any area of residual stenosis identified at this stage prompts targeted additional decompression before closure.

Hemostasis is achieved with bipolar electrocautery and thrombin-soaked absorbable hemostatic material as needed. The wound is irrigated copiously. Closure is performed in standard layered fashion with reapproximation of the paraspinal musculature, complete fascial closure, subcutaneous layer closure, and skin closure per surgeon preference. A subfascial drain may be placed at the surgeon’s discretion. The overall technique described in Steps 1 through 6 is specifically designed to be taught to neurosurgical residents and is summarized in Table 1. Each step has a defined safety rationale, a teachable skill, and a navigation checkpoint that allows the attending to verify the resident’s work in real time. For experienced neurosurgeons, the technique offers a structured and rapid alternative to Kerrison-based piecemeal laminectomy, with navigation providing objective decompression verification at each level.

**Table 1:** Operative steps summary Summary of the six operative steps, instruments, navigation checkpoints, and primary safety considerations for the bilateral troughing laminectomy technique.

Step	Name	Primary Instrument	Navigation Checkpoint	Key Safety Consideration	Applicable To
1	Lateral Boundary Confirmation	Navigated probe	Yes: confirms lateral drilling boundary at each level	Prevents inadvertent facetectomy before any drilling begins	All cases
2	Bilateral Trough Creation	3 mm matchstick burr	Yes: confirms lateral extent does not encroach on facet or pedicle	Maintains bony buffer over dura; avoids facet violation	All cases
3	Eggshell Thinning	5 mm diamond burr	Yes: confirms bilateral trough depth and eggshell completion	Prevents inadvertent dural penetration during final cortical thinning	All cases
4	Lamina Removal	Heavy Mayo scissors, Kocher clamp	No	Trough geometry constrains fracture plane; protects dura during elevation	All cases
5	Ligamentum Flavum Resection and Lateral Recess Decompression	Woodson elevator, Kerrison rongeurs, diamond drill	Yes: confirm undercutting of articular process no more than 30% of articular surface	Protects dural sleeve and nerve root during flavectomy and undercutting	As needed
6	Final Navigation Verification	Navigation system	Yes: lateral wall, facet preservation, cranio-caudal extent	Identifies residual stenosis before closure; prompts targeted additional decompression	All cases

Postoperative Outcome

The estimated blood loss was 30 mL. The bilateral troughing and en bloc laminectomy portion of the procedure, spanning L3-5, was completed in approximately 15 minutes. The patient was neurologically intact on immediate postoperative assessment, with preserved motor strength and intact sensation bilaterally. He reported significant resolution of his preoperative bilateral leg pain. Residual axial back pain at the incision site was rated 3/10 on postoperative day one. He was mobilized on postoperative day one with physical therapy and was discharged home in stable condition on postoperative day three without complication. At one-month postoperative follow-up, he reported a meaningful improvement in lower extremity symptoms compared to his pre-operative baseline with a new pain score of 3/10. Longer-term follow-up and validated patient-reported outcome measures were not captured for this single illustrative case and represent a planned component of the prospective series described in the Limitations.

## Discussion

We describe a structured, reproducible approach to lumbar laminectomy centered on a staged bilateral troughing technique using a 3-mm matchstick burr for outer cortical trough creation and a 5-mm diamond burr for final inner cortical thinning, with intraoperative navigation providing anatomical confirmation at defined checkpoints. The primary contribution of this technique is the formalization of a structured, safety-focused operative sequence designed to be taught to neurosurgical residents while remaining efficient for experienced surgeons. The staged burr strategy reduces the risk of inadvertent dural injury inherent to piecemeal Kerrison-based laminectomy by maintaining a bony buffer until controlled lamina elevation is performed. Each step carries a defined safety rationale, a teachable skill, and a navigation checkpoint that allows objective verification of anatomical boundaries.

The rationale for a staged burr strategy is grounded in the complementary cutting characteristics of the two instruments employed. The matchstick burr, with its lateral cutting profile, is efficient for trough creation along a defined line and allows rapid reduction of the outer laminar cortex without the axial penetration risk of an end-cutting instrument. As the inner cortex is approached and the margin between the burr tip and the underlying dura narrows, this efficiency advantage becomes a liability: the same lateral cutting force that makes the matchstick burr well-suited to open bone can result in inadvertent dural snagging at depth. The diamond burr addresses precisely this risk. Its irrigated abrasive surface removes bone progressively and predictably without the mechanical cutting edge that can engage the dura or underlying epidural structures. The holding direction of the drill away from the dura and the cranial caudal painting movement removes the final layers without plunging. The practice of transitioning from a cutting to a diamond burr for final cortical thinning is well established in posterior fossa and skull base surgery; its application to the lumbar posterior arch is a natural and logical extension of this principle [13].

Facet joint preservation is among the most clinically consequential determinants of long-term outcomes following lumbar decompression without fusion. Graded biomechanical studies - both cadaveric and finite-element - consistently demonstrate that facet resection beyond approximately one-third (30%) of the articular surface destabilizes the lumbar motion segment, with instability worsening progressively as resection extends further [1,10-12]. Cadaveric work established that medial facetectomy alone does not significantly affect spinal stability, whereas total facetectomy, even performed unilaterally, reliably produces instability in axial rotation and extension [1]. Finite-element analyses corroborate this threshold: bilateral resection of 30%-45% of the articular surface elevates anteroposterior and mediolateral mobility and raises intradiscal pressure, effects that compound at greater resection depths [10,11]. Decompression limited to one-third of the joint preserves segmental biomechanics comparable to the intact state [12]. The medial facet border, therefore, represents not merely an anatomical landmark but a functional boundary with direct implications for the durability of the decompression. Navigation-based confirmation of trough position relative to this boundary provides an objective, real-time check that tactile and visual estimation alone cannot reliably replicate, particularly in cases with high body mass index, multilevel exposure with distorted anatomy, or revision surgery where normal tissue planes have been altered by prior scarring.

Our group previously described a handheld osteotome approach to single-unit laminectomy, validating the technique in a cadaveric model and demonstrating its clinical application in an illustrative case [8]. The troughing approach described here uses conventional high-speed burrs universally available in spinal operating rooms, adds a navigation-confirmed anatomical boundary framework, and formalizes the transition from cutting to diamond burr to address dural safety near the inner cortex. Together, these techniques demonstrate the feasibility and advantages of non-piecemeal posterior arch removal for degenerative lumbar stenosis. The trough lines serve as controlled fracture planes along which the eggshell lamina separates during elevation, producing a clean en bloc specimen without the thermal or vibrational energy transmitted by an oscillating saw described in prior studies [7]. Importantly, en bloc ligamentum flavum resection has been shown to eliminate iatrogenic CSF leaks entirely (0% vs. 6.4% with piecemeal resection), further supporting the safety advantage of a controlled single-unit removal strategy [15].

The role of intraoperative navigation as an adjunct to decompression, as distinct from its established role in instrumentation, has attracted increasing interest. Postoperative CT studies have demonstrated residual stenosis in a substantial proportion of lumbar decompression cases, and intraoperative imaging allows real-time identification and correction of incomplete decompression before the patient leaves the operating room [16]. Visible-light and machine-vision navigation systems have demonstrated rapid, reliable registration achievable without radiation exposure, with case series reporting reduced operative times compared to fluoroscopy-guided approaches when navigation is integrated into the workflow [17]. Critically, in our technique, navigation functions as a confirmatory adjunct rather than a primary guide. The surgeon directs the decompression based on anatomical knowledge, direct visualization, and tactile feedback; navigation verifies that the result is anatomically correct at each defined checkpoint. This distinction is important: it preserves the surgeon’s operative judgment while capturing the precision benefit of objective anatomical verification, and avoids the automation complacency that can arise from over-reliance on image guidance. Practically, this is enforced by the structure of the technique itself: each navigation checkpoint is triggered by a defined anatomical milestone that the surgeon has already reached through direct work, not by a navigation prompt. The surgeon decides when to verify; navigation confirms or corrects. This sequence maintains the surgeon’s situational awareness and prevents passive dependence on image guidance that can erode operative skill over time.

A critical and underappreciated strength of this technique is its suitability as a structured teaching tool for neurosurgical residents. Lumbar laminectomy is taught largely through apprenticeship with minimal standardization of the decompressive sequence. The six-step troughing technique described here transforms this experience-dependent procedure into a formal curriculum: each step has an explicit anatomical objective, a defined safety principle, and a navigation checkpoint that allows an attending to objectively verify trainee progress in real time. Learning curves for structured spine procedures have been shown to plateau at approximately 30 cases with a stepwise protocol, compared with far longer apprenticeship-based learning [18]. The tactile feedback of matchstick burr trough creation, the visual cue of the cancellous-to-cortical transition, and the diamond burr eggshell endpoint together constitute a progressive skill sequence that builds both anatomical understanding and operative judgment. For residency programs at academic medical centers where intraoperative navigation is already standard for pedicle screw placement, extending this infrastructure to the decompressive portion adds no capital cost and directly benefits trainees learning to operate safely within defined anatomical boundaries.

Preservation of the posterior bony architecture, specifically the pars interarticularis and facet joint complex, is a fundamental principle that this technique is designed to respect. Biomechanical data support limiting facetectomy to no more than approximately one-third (30%) of the articular surface to minimize the risk of postoperative instability [1,10-12]. Resection beyond this threshold produces progressive and clinically meaningful increases in segmental motion. Pars interarticularis resection should similarly be limited to no more than 50%, as removal beyond this point significantly compromises structural integrity. The anatomic margins available for safe decompression also vary by level: cadaveric and imaging studies demonstrate that the ratio of pars interarticularis distance to spinal canal width is narrower at upper lumbar levels (L1-L3) than at L4-L5, reflecting the relatively larger pars interarticularis at L5 compared with more rostral segments [14]. These level-specific differences underscore why a fixed millimeter threshold for bony resection cannot be uniformly applied across the lumbar spine, and why real-time navigation feedback is particularly valuable in guiding safe resection margins at upper lumbar levels.

Incidental durotomy warrants explicit discussion as an intraoperative complication of lumbar decompression. The Spine Patient Outcomes Research Trial (SPORT) trial database, representing the largest prospective analysis of its kind, documented an incidental durotomy rate of approximately 9% across lumbar decompression cases. Desai and colleagues demonstrated that, while durotomy was associated with significant perioperative burden, including a 29% increase in operative time, 85% increase in estimated blood loss, and 39% increase in length of hospital stay, incidental durotomy carried no significant effect on patient-reported outcomes at four-year follow-up, including Oswestry Disability Index scores, leg pain, or patient satisfaction [19]. These findings are reassuring with respect to long-term prognosis but underscore that incidental durotomy remains a clinically meaningful perioperative event with real implications for immediate resource utilization and patient experience. The staged troughing technique described here, which maintains a cortical buffer until controlled laminar elevation and employs the diamond burr for final inner cortical thinning precisely to avoid plunge-type dural penetration, is designed to minimize this risk; nonetheless, surgeons should counsel patients regarding the possibility of this complication and its perioperative implications.

Limitations

Several limitations of this technique and the present report warrant acknowledgment. First, the approach requires familiarity with high-speed burr instrumentation and the tactile discrimination needed for eggshell thinning near the inner cortex; surgeons trained primarily in rongeur-based laminectomy may find the adoption curve non-trivial, and structured cadaveric training is advisable before clinical implementation. Second, navigation adds equipment cost and, for CT-based platforms, radiation exposure to the patient and operative team. In our case, we elected to use a visible-light surface navigation system to mitigate the radiation concern and reduce operative time substantially. Notably, however, navigation is not a limiting factor for wide adoption: neurosurgical residency programs are predominantly affiliated with academic medical centers and tertiary care facilities that routinely employ intraoperative navigation for pedicle screw placement. Extending this same infrastructure to decompressive procedures adds no new capital requirement and is immediately actionable at most training institutions.

The present report describes an operative technique and a single illustrative case, and is not intended as a comparative outcome study. It does not provide comparative outcome data, quantitative decompression metrics across a cohort, or a formal learning curve analysis; the perioperative outcomes reported (blood loss, operative time, length of stay, and one-month follow-up) should be interpreted as proof-of-concept feasibility data rather than as evidence of superiority over conventional laminectomy. These limitations are inherent to the technique description format and define the scope of the prospective work required to establish the clinical utility of this approach. Future studies should prospectively (1) capture dural sac cross-sectional area before and after decompression using standardized MRI measurement protocols and report postoperative DSCA at each operated level, (2) quantify facet preservation on postoperative CT as the proportion of articular surface retained bilaterally, (3) record trough creation and total decompression times per level to characterize the efficiency profile of the technique, reporting decompression time per level separately from total operative time, (4) capture validated patient-reported outcome measures including the Oswestry Disability Index and visual analogue scale scores at standardized follow-up intervals, and (5) compare patient-reported outcomes against a matched cohort undergoing conventional piecemeal laminectomy. A future prospective case series would provide sufficient power for preliminary feasibility and safety endpoints, with a formal comparative study as the logical subsequent step.

## Conclusions

Navigation-assisted bilateral troughing laminectomy using a staged matchstick-to-diamond burr technique provides a structured, reproducible, and teachable approach to lumbar decompression. The technique formalizes the decompression sequence into defined anatomical checkpoints consisting of medial facet border confirmation, trough depth verification, eggshell completion, lamina removal, lateral recess undercutting, and final decompression verification. This reduces reliance on purely subjective estimation at each critical decision point, especially when training neurosurgical residents. The sequential burr strategy balances operative efficiency with dural safety, en bloc removal provides consistent and immediate thecal sac visualization, and navigation serves as a practical confirmatory adjunct that enhances precision without disrupting operative flow. This approach merits evaluation in prospective cohort studies to establish its decompression adequacy, efficiency profile, and complication rate relative to conventional techniques.
